# Effect of Calcium on Absorption Properties and Thermal Stability of Milk during Microwave Heating

**DOI:** 10.3390/ijms19061747

**Published:** 2018-06-13

**Authors:** Yejun Wu, Daming Fan, Feng Hang, Bowen Yan, Jianxin Zhao, Hao Zhang, Wei Chen

**Affiliations:** 1 State Key Laboratory of Dairy Biotechnology, Technology Center, Bright Dairy & Food Co., Ltd., Shanghai 200436, China; 6170112090@stu.jiangnan.edu.cn (Y.W.); 6160112071@vip.jiangnan.edu.cn (F.H.); weichen@jiangnan.edu.cn (W.C.); 2 State Key Laboratory of Food Science and Technology, Jiangnan University, Wuxi 214122, China; bwyan1@126.com (B.Y.); zhanghao@jiangnan.edu.cn (H.Z.); 3 School of Food Science and Technology, Jiangnan University, Wuxi 214122, China; 4 National Engineering Research Center for Functional Food, Jiangnan University, Wuxi 214122, China; 5 Collaborative Innovation Center of Food Safety and Quality Control in Jiangsu Province, Wuxi 214122, China

**Keywords:** calcium, milk, microwave heating, absorption properties, thermal stability

## Abstract

During heating, there are a lot of physical and chemical changes in milk components, which are mainly reflected in the changes of proteins. Calcium ions in milk react with proteins to precipitate or form gels, and the thermal stability of milk is affected by the type and content of calcium. In this study, different calcium-fortified milk systems were treated by rapid conventional heating (RCV) and microwave heating (MV) to evaluate the effects of forms and concentration of calcium in liquid milk on microwave absorption properties and thermal stability of milk. It was found that the concentration of calcium ions on microwave energy absorption is not a significant influence, while the forms affected the systems dramatically. The thermal stability of milk during MV is remarkably affected by the forms of calcium ions. When adding ionized calcium, the calcium-fortified milk systems had poor thermal stability and severe agglomeration of protein, while the addition of milk calcium had little effect and was almost free from protein coagulation. It could be speculated that the metal ions in the microwave field could create a strong vibration that could trigger protein agglomeration through the combination of the surrounding casein phosphorylates.

## 1. Introduction

Milk is one of the oldest natural beverages, which has a high nutritional value and is the best source of nutrition for new life. Milk proteins are mainly composed of casein and whey proteins, including all the essential amino acids. 100 g of common milk contains about 100 mg of calcium. A significative amount of calcium is present as a colloidal form of casein calcium phosphate, which is more likely to be absorbed by the body than the calcium found in vegetables and fruits, thereby calcium-fortified foods are favored by most governments and health organizations.

Heating milk has always been a key process in the dairy industry. During heating, there are a lot of physical and chemical changes in milk components [1]. When heating to greater than 60 °C, it causes some significant changes such as whey protein denaturation, changes in casein micelles, reactions between denatured whey proteins and casein micelles, reactions between lactose and proteins, changes in milk globulin membrane proteins, conversion of soluble forms of calcium, magnesium, phosphorus, and other elements to colloidal states, the reduction of pH, and loss of vitamins and bioactive substances. The thermal stability of milk is affected by the type of calcium salt and the milk system [2,3,4,5,6].

Microwave heating is a typical fast heating method that works by alternating cycle changes in the microwave electric field and magnetic field. The polarity orientation of the dipole in milk macromolecules varies with the change of the external electromagnetic field, and under the action of the frequency modulation (FM) electromagnetic field of 2.45 GHz, the polar molecules rotate several billion times per second. The sharp friction, collision, vibration, extrusion, and other effects between the rotating molecules produces heat, so parts of the material simultaneously gain heat. Both the target activation hypothesis and thermal agitation hypothesis focus on the induction of the dielectric enhancement effect of the dielectric sensing component in the liquid phase of the system. In the microwave field, the calcium ions have high dielectric response characteristics. The molecular vibrations during heating can affect the interaction of the phosphate groups on the surface of caseins with the calcium ions and thus contribute to protein agglomeration [7,8].

In this study, we investigated how different forms and concentrations of calcium affected the absorption properties and thermal stability of milk and analyzed the differences between milk heated by rapid heat conduction and microwave heating to learn how the quality of calcium-fortified milk is affected by the different heating methods. The results could provide information about the changes in milk during heat processing and may also provide an important theoretical basis for the extensive application of microwave ultra-high temperature (UHT) sterilization in the future.

## 2. Results and Discussion

### 2.1. The Basic Components of Milk

The components of milk are very complex and contain more than 3000 kinds of compounds, the basic being fat, protein, lactose, inorganic salt, vitamins, and water. Under normal conditions, the components are relatively stable. The basic components of blank milk system are shown in Table 1.

**Figure 1:**
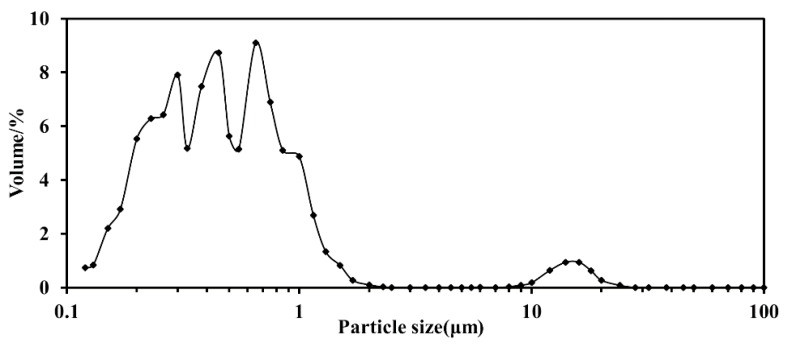
The particle size distribution of blank milk system.

Particle size analysis is a method that can quickly analyze the thermal stability of a system. If the particle size distribution of the system is Gaussian, i.e., the mean, median, and frequency are in the same position, the system is in a relatively stable state; conversely, there are instability factors in the system [9]. The particle size distribution of blank milk samples is shown in Figure 1 and Table 2.

### 2.2. Effects of Calcium on the Dielectric Properties and Microwave Absorption Properties of Milk

#### 2.2.1. Dielectric Properties of the Calcium-Fortified Milk Systems

The dielectric property is an inherent response characteristic of a bound charge within a molecule to an applied electric field. The dielectric constant ε′ and dielectric loss factor ε′′ are commonly used to express the parameters of dielectric properties. The dielectric constant ε′ represents the ability of the calcium-fortified milk systems to store electromagnetic waves because of the electrical properties of the system that affect energy absorption and transfer, and the dielectric loss factor ε′′ represents the ability of the systems to consume electrical energy in the form of heat [10]. Milk is an inhomogeneous colloidal dispersion system. The protein colloids and fat particles are dispersed in water that contains salts, lactose, and whey proteins. For nonhomogeneous substances, the different phases have unique dielectric parameters. Electric charges can accumulate at the different phase boundaries, causing the two phases to polarize at the interface. Changes in the dielectric parameters of milk are mainly caused by the dipoles in the inhomogeneous material, the polarization of electrons and atoms and the Maxwell-Wagner effect [11,12].

**Figure 2:**
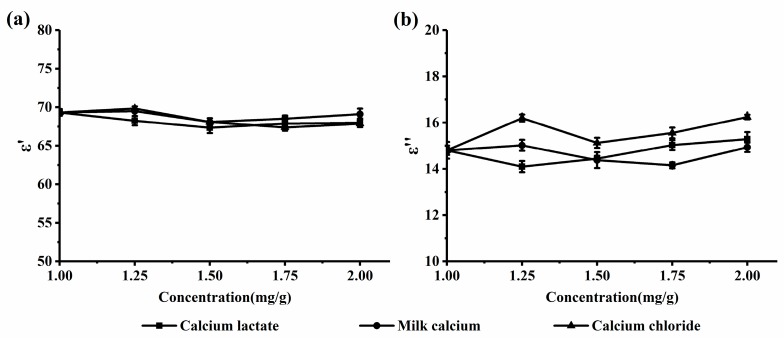
(**a**) The dielectric constant ε′ and (**b**) dielectric loss factor ε′′ of the calcium-fortified milk systems.

To study the effects of different forms and concentrations of calcium on the dielectric properties of milk, here, we chose three sources of calcium: calcium chloride, calcium lactate, and milk calcium. The calcium concentrations were 1 (blank), 1.25, 1.5, 1.75, and 2 mg/g. The changes of the dielectric constant ε′ and the dielectric loss factor ε′′ at 2.45 GHz in different milk systems are shown in Figure 2. As shown in Figure 2a, the ε′ did not change significantly with different forms of calcium and with the increase in calcium concentration at room temperature. As shown in Figure 2b, the ε′′ fluctuated slightly with the increase in calcium concentration and the fluctuations of calcium chloride and milk calcium were contrary to calcium lactate, moreover, the ε′′ of the calcium chloride was maximal and fluctuant obviously, while the ε′′ of the calcium lactate and milk calcium were closer and had a couple of small fluctuations. That is, with the increase in calcium concentration, the ability of the systems to absorb and store the microwave energy in the microwave field did not change, but the ability to convert the absorbed microwave energy into heat energy fluctuated slightly. In addition, the means by which the different forms of calcium affected the ability of the system to absorb and store microwave energy were not obvious but had an effect on the ability of the system to convert the absorbed microwave energy into heat energy; more specifically, the ionized form of calcium, calcium chloride, had the greatest influence.

#### 2.2.2. Microwave Absorption Properties of the Calcium-Fortified Milk Systems

**Figure 3:**
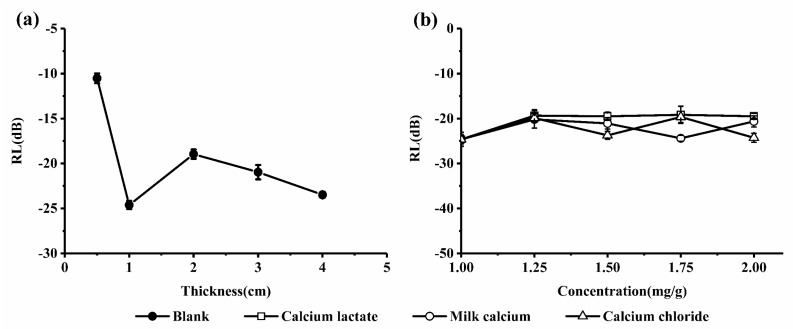
The reflection loss (RL) of (**a**) the blank milk at different thicknesses and (**b**) the calcium-fortified milk at a thickness of 1 cm.

The response of a material in a microwave field cannot be reflected only by its complex permittivity, as it can also be affected by the impedance matching problem and others. The microwave absorption properties of the material are the comprehensive performance of its dielectric properties, its magnetic properties and the macroscopic morphology [13]. Reflection loss (RL) is an important parameter to evaluate the absorption performance of the material, and the RL of the material reflects its absorption, release and loss ability of the microwave [14,15,16]. We measured the RL of the blank milk systems at different thicknesses to observe the absorption properties. As shown in Figure 3a, the RL of the blank at 2.45 GHz first showed a tendency to increase and then decreased with the thickness increasing and the systems reached a maximum absorption of −24.627 dB at a thickness of 1 cm. Therefore, it can be concluded that the absorption properties of the material were not linearly related to its thickness and there was an optimal thickness at the specified frequency of 2.45 GHz. From above, a standard thickness of 1 cm was chosen to conduct the follow-up studies.

It can be seen from Figure 3b that the calcium concentration increased from 1 mg/g to 2 mg/g at 2.45 GHz, and the RL of the systems did not change significantly, while the different forms of calcium production affected the system in the following ways: (i) in the range of 1 to 1.25 mg/g, with the increase of calcium concentration, the effect of calcium chloride, calcium lactate, and milk calcium on the systems was consistent, and the RL decreased slightly; (ii) in the range of 1.25 to 2 mg/g, the effect of the three forms of calcium on the systems differed as the calcium concentration increased. Calcium lactate almost had no effect and the RL (in dB). In contrast, calcium chloride and milk calcium made RL fluctuate being the effect of calcium chloride greater.

### 2.3. The Thermal Stability of the Calcium-Fortified Milk Systems under the Microwave Field

#### 2.3.1. Comparison of the Microwave Heating and Rapid Conventional Heating Curves

During MV, the thermal and non-thermal effects were simultaneously applied to the material. In this study, multistage MV programs were used to match the RCV curves to achieve similar heating rates so that we could study the non-thermal effects of the microwave on the systems. The root mean square error (RMSE) was calculated according to Equation (1) to evaluate the accuracy between the MV and RCV data [17].

(1) $$\mathrm{RMSE}=\sqrt{\frac{1}{N}\sum_{n=1}^{N}{\left(T_{MV}-T_{RCV}\right)}^{2}}$$

**Figure 4:**
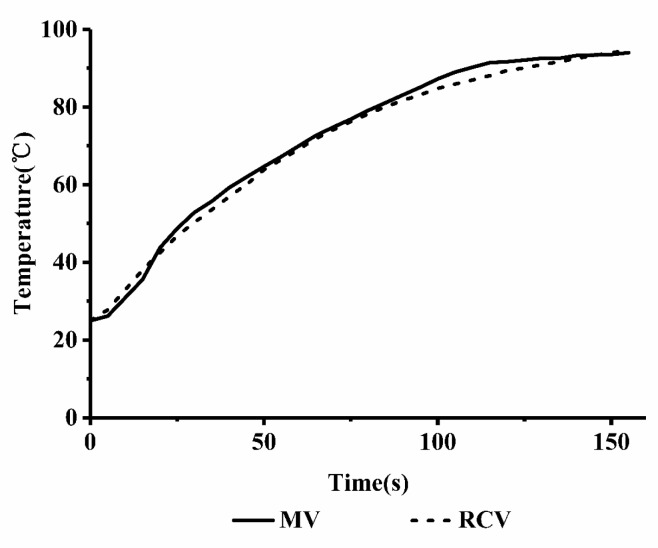
Comparison of the microwave heating(MV) and rapid conventional heating (RCV) curves.

As shown in Figure 4, the RMSE value was 1.77, representing the difference in the average temperatures of RCV and MV. This result indicated that the MV curve fits well with RCV processing.

#### 2.3.2. The Thermal Stability of the Milk during the Heating Process

Under the same MV conditions, the blank and 1.5 mg/g milk calcium-fortified milk systems were heated to various temperatures and their particle size changed significantly, as shown in Table 3. With the increase of temperature, the size of particles in two milk systems shifted to be larger and the percentage of particles (10–100 μm) increased to 15.72% and 14.51%, respectively, when the systems were heated to 95 °C by MV. This could be that the heating temperature was too high, causing intense aggregation of milk protein particles and denaturation of whey protein, and leading to the whey protein to form protein polymers with itself or with casein. Subsequently, we conducted experiments at 95 °C to investigate the effects of the different calcium forms, concentrations, and heating methods on the thermal stability of milk.

#### 2.3.3. The Effects of Different Calcium Forms on the Thermal Stability of Milk

**Figure 5:**
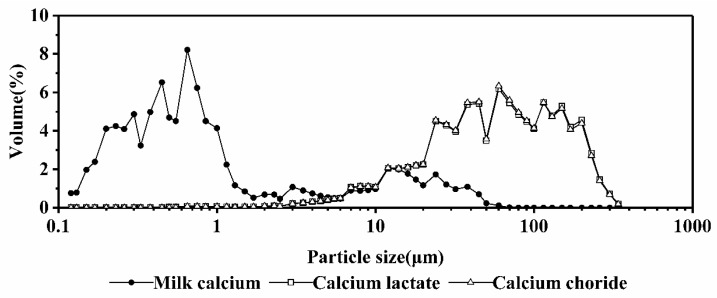
The particle size distribution of different calcium-fortified milk systems at 95 °C.

Figure 5 shows the particle size distribution for the milk calcium, calcium lactate and calcium chloride fortified milk systems at 95 °C. In the milk calcium-fortified systems, the particles were mainly distributed in 0.1 to 1 μm, and 0.82 μm were the most common. However, in the case of calcium lactate and calcium chloride, the particles were mainly distributed in 10 to 340 μm and a few in 1.0 to 10 μm. These showed that the effect of milk calcium on the thermal stability of milk systems was little and had no protein aggregation. In contrast, calcium chloride and calcium lactate had a great influence on the systems, forming larger particles and substantial protein aggregation.

The addition of milk calcium made the systems’ structure more uniform. It also created a buffering effect so that the pH of the systems remained stable and had little effect on the thermal stability [18]. However, the addition of ionic calcium in the form of either calcium chloride or calcium lactate could have altered the original salt balance of the milk systems. We observed the following effects: colloidal calcium phosphate dissolved; casein lost its electrostatic charge and agglomerated; fat was constantly aggregated to form larger fat particles; and calcium precipitated after polymerization to form larger particles. These effects showed that the calcium chloride and calcium lactate fortified milk systems were less thermostable because internal polymerization occurred and the fat granules and casein particles that were originally small gradually polymerized to form more large particles. The large fat granules and casein particles resulted in increased agglomeration [19].

#### 2.3.4. The Effects of Different Calcium Concentrations on the Thermal Stability of Milk

**Figure 6:**
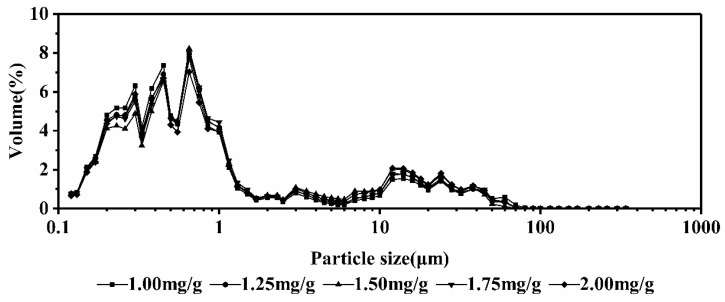
The particle size distribution of different concentrations of milk calcium-fortified milk systems at 95 °C.

For the milk calcium-fortified systems, as shown in Figure 6, the particle sizes were consistently distributed in 0.1 to 1 μm, indicating that different calcium concentrations had little effect on the thermal stability of the systems under the microwave field. Also, there was no protein aggregation.

#### 2.3.5. The Effects of Different Heating Methods on the Thermal Stability of Milk

**Figure 7:**
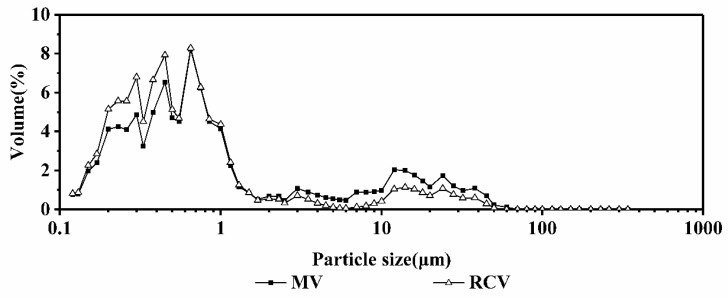
The particle size distribution of different concentrations of milk calcium-fortified milk systems at 95 °C.

For the 1.5 mg/g milk calcium-fortified systems, as shown in Figure 7, no significant differences were seen in the particle size distribution curves by MV or RCV, and the particle sizes were roughly distributed in 0.1 to 1 μm, indicating that the different heating methods had little effect on the thermal stability of the milk systems.

## 3. Materials and Methods 

### 3.1. Materials

Pure milk and milk calcium (Bright Dairy & Food Co., Ltd., Shanghai, China); Anhydrous calcium chloride and calcium lactate (Sinopharm Chemical Reagent Co., Ltd., Shanghai, China).

### 3.2. Preparation of Calcium-Fortified Milk Systems

Referring to the calcium content in high-grade calcium milk and its preparation method (Patent number: CN 103651846 A) [20], calcium concentrations of 1 (blank), 1.25, 1.5, 1.75, and 2 mg/g were chosen.

A 100-g calcium-fortified milk system was prepared for each experiment. The amounts of calcium chloride, calcium lactate, and milk calcium added to the system are shown in Table 4.

### 3.3. Determination of Dielectric Properties

The ε′ and ε′′ values were measured using a vector network analyzer (E5071C, Agilent, Santa Clara, CA, USA) with an open-ended coaxial line, connected to a high-temperature probe (85070E, Agilent) [21,22]. The probe was calibrated with air, a short circuit, and water, respectively. Each sample was measured in triplicate.

### 3.4. Determination of Reflection Loss (RL)

**Figure 8:**
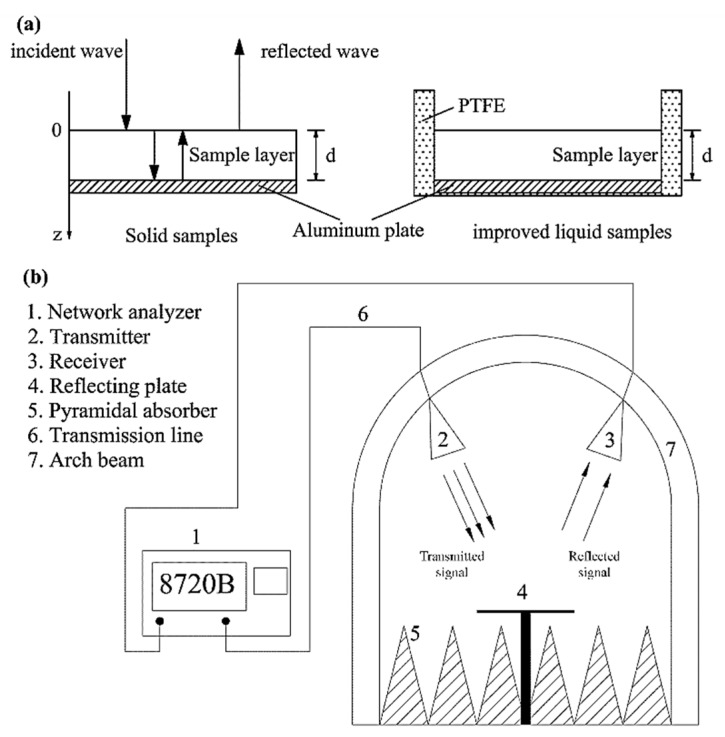
The improved specialized container for liquid samples (**a**) and a schematic diagram of the arch method testing system (**b**).

The RL versus frequency for liquid materials was improved based on the arch method by adding polytetrafluoroethylene (PTFE) as a wall material on an aluminum plate, to make a specialized container (Figure 8a). The testing system of the improved arch method included a vector network analyzer (HP8720B, Hewlett Packard Co., Palo Alto, CA, USA) and standard horn antennas in an anechoic chamber as a function of frequency from 2.3 to 2.5 GHz (Figure 8b) [23]. In this testing system, the signal was emitted from the transmitter and reflected by a metal reflecting plate, then the reflected signal was received by the receivier and passed into the vector network analyzer, so that a certain frequency established a power reference. The difference between the received power of the metal plate and the sample to be measured was converted into a reflection loss.

### 3.5. Rapid Conventional Heating Method

**Figure 9:**
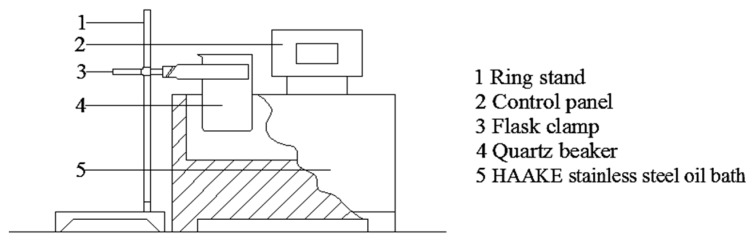
The device of rapid conventional heating: a HAAKE stainless steel oil bath.

In terms of heating conditions, a HAAKE stainless steel oil bath (AC200, Thermo Scientific, Waltham, MA, USA) was used for RCV while the temperature was monitored online using a thermocouple thermometer (Shifu Instrument Co., Ltd., Wuhu, China) [24], as shown in Figure 9. A 50 g of blank sample was weighed into a quartz beaker and set it at 195 °C, a temperature determined by a previous experiment, kept the stirrer open (45 kr/min) during heating to ensure that the sample was heated evenly and reliably. To achieve a rapid heat transfer, the control time was set to less than 3 min. The milk was heated to the specified temperature (45, 65, 95 °C) using the temperature probe to detect the internal temperature. The real-time temperature of the system was recorded to plot a temperature curve as the reference for the microwave heating conditions. To prevent the sample temperature from rising past the specified limit, the sample was immediately cooled in an ice bath [25].

### 3.6. Microwave Heating Method

**Figure 10:**
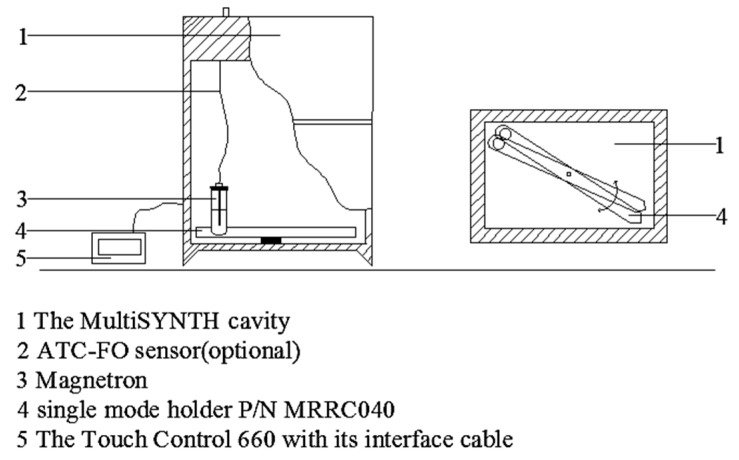
The device of Microwave heating: a MultiSYNTH microwave synthesizer.

An advanced MultiSYNTH microwave synthesis platform (MultiSYNTH, Milestone, Sorisole, Italy) was used to simulate the RCV heating curves [26], as shown in Figure 10. A 4 g of the sample was weighed into a quartz tube that was 1.2 cm in diameter. The tube was placed in a polyester sleeve without electromagnetic absorption and then placed into the cavity sample pool of the MultiSYNTH microwave synthesizer (Milestone, Sorisole, Italy). The synthesizer settings were microwave single-mode processing, a microwave frequency of 2.45 GHz and the vibration frequency at 10%. The MultiSYNTH system detected the real-time temperature of the test tube using the infrared temperature probe. By adjusting the vibration frequency of the sample pool, the microwave synthesizer allowed the sample to receive uniform radiation and to have a uniform temperature distribution. When the temperature reached the preset temperature, the sample was immediately cooled in an ice bath. Multiple replications were performed during the preliminary stages to determine the power necessary to match the oil bath. The MV procedure can be seen in Table 5.

### 3.7. Analysis of Milk Composition

A Milko-ScanTM FT1 multifunction dairy analyzer (Foss, Hillerød, Denmark) was used to compare the differences between the compositions of the calcium-milk composite system when treated by RCV or by MV. The components tested included fat content (FAT, %), crude protein content (Cru.Prot, %), true protein content (Tru.Prot, %), lactose content (Lactose, %), solids non-fat content (SnF, %), total solids content (Ts, %), freezing point (FPT, °C).

### 3.8. Analysis of Particle Size

A BT-9300H laser particle size distribution instrument (Bettersize Instruments Co., Ltd., Dandong, China) with a BT-600-type circulating dispersion pump was used. The samples which were placed at 20 ± 1 °C for 30 min before testing consisted of controls (blanks), milk samples treated via RCV and MW. All samples were not diluted. A moderate number of samples were added to the sample cell with water as the dispersion medium, opened the ultrasonic dispersion and collected measurements at a rotational speed of 320 rpm to obtain the particle size distribution.

### 3.9. Data Analysis

All statistical analyses were performed with Origin 2017 and SPSS. The data are presented as mean ± SD for each group. The differences between the mean values of the groups were analyzed using a one-way variance analysis with Duncan’s multiple range tests. A *p* value of less than 0.05 was considered to indicate statistical significance.

## 4. Conclusions

We investigated the effects of different forms and concentrations of calcium on the microwave absorption properties and the thermal stability of milk. The results showed that the effect of different forms and concentrations of calcium on the absorption of microwave energy was not significant, but the presence of ionic calcium had a relatively great influence. Compared with RCV and unheated, the calcium-fortified milk systems treated by MV had no significant loss of nutrients. Furthermore, the milk systems with added ionized calcium had poor thermal stability and severe aggregation of proteins during MV, while the addition of milk calcium had little effect and there was almost no protein aggregation in the systems.

In fast-paced modern life, the quality of rejuvenating liquid milk is a focus for consumers. Microwave heating is a typical fast method for reheating, but its safety has always been controversial. This study investigated the microwave heating process of calcium-fortified milk systems, which can provide a theoretical basis for the study of the mechanisms that underlie the dielectric intervention effect of calcium-induced milk proteins in microwave fields and lay a foundation for the development of microwave sterilization technology.

## Figures and Tables

**Table 1:** The basic components of blank milk system. SnF, Ts, FPT

Sample	Fat/%	Cru.Protein ^1^/%	Tru.Protein ^2^/%	Lactose/%	SnF ^3^/%	Ts ^4^/%	FPT ^5^/°C	Calcium/%
Blank milk	3.50 ± 0.19	3.07 ± 0.30	2.91 ± 0.21	4.62 ± 0.11	8.54 ± 0.31	12.08 ± 0.30	0.495 ± 0.14	0.10 ± 0.03

^1^ Cru.Protein means crude protein content, ^2^ Tru.Protein means true protein content, ^3^ SnF means solids non-fat content, ^4^ Ts means total solids content, ^5^ FPT means freezing point.

**Table 2:** The particle size distribution of blank milk system.

Sample	The Percentage of Particles in Different Distribution/%		
	0.1–1 μm	1–10 μm	10–100 μm
Blank milk	90.95 ± 0.23	5.55 ± 0.31	3.50 ± 0.56

**Table 3:** The percentage of different particle sizes in the different milk systems at various temperatures.

Samples	Temperature	0–1 μm	1–10 μm	10–100 μm
Blank milk systems	25 °C	90.95 ± 0.23	5.55 ± 0.31	3.50 ± 0.56
	45 °C	82.28 ± 0.59	7.28 ± 0.42	9.44 ± 0.60
	65 °C	76.33 ± 0.88	10.66 ± 0.74	13.01 ± 0.62
	95 °C	71.69 ± 0.45	12.59 ± 0.51	15.72 ± 0.54
milk calcium-fortified systems	45 °C	69.84 ± 0.69	15.62 ± 0.51	14.54 ± 0.72
	65 °C	69.83 ± 0.54	15.65 ± 0.72	14.52 ± 0.49
	95 °C	70.35 ± 0.73	15.14 ± 0.93	14.51 ± 1.21

**Table 4:** Amounts of calcium chloride, calcium lactate and milk calcium added to the system.

Calcium Content	Calcium Chloride	Calcium Lactate	Milk Calcium
1.00 mg/g	0	0	0
1.25 mg/g	0.0694 g ± 0.0002	0.1927 g ± 0.0001	0.0975 g ± 0.0002
1.50 mg/g	0.1387 g ± 0.0003	0.3854 g ± 0.0000	0.1951 g ± 0.0002
1.75 mg/g	0.2081 g ± 0.0001	0.5781 g ± 0.0002	0.2926 g ± 0.0000
2.00 mg/g	0.2775 g ± 0.0001	0.7708 g ± 0.0003	0.3902 g ± 0.0001

**Table 5:** Microwave heating procedure for a 4 g sample of the calcium-fortified milk systems.

Heating Stages	Power/W	Time/s
First stage	72	15
Second stage	39	50
Third stage	33	45
Fourth stage	31	45
